# Virtual monochromatic spectral imaging versus linearly blended dual-energy and single-energy imaging during CT-guided biopsy needle positioning: Optimization of keV settings and impact on image quality

**DOI:** 10.1371/journal.pone.0228578

**Published:** 2020-02-10

**Authors:** T. D. Do, J. Heim, C. Melzig, D. F. Vollherbst, H. U. Kauczor, S. Skornitzke, C. M. Sommer

**Affiliations:** 1 Clinic for Diagnostic and Interventional Radiology, University Hospital Heidelberg, Heidelberg, Germany; 2 Department of Neuroradiology, University Hospital Heidelberg, Heidelberg, Germany; 3 Clinic for Diagnostic and Interventional Radiology, Klinikum Stuttgart, Stuttgart, Germany; National University of Singapore, SINGAPORE

## Abstract

**Objectives:**

To compare image quality and metal artifact reduction between virtual monochromatic spectral imaging (VMSI), linearly blended dual-energy (DE) and single-energy (SE) images, each with and without dedicated iterative metal artifact reduction (iMAR) for CT-guided biopsy.

**Materials and methods:**

A biopsy trocar was positioned in the liver of six pigs. DE (Sn140/100kV_p_) and SE (120kV_p_/200mAs) acquisitions were performed with equivalent dose. From dual-energy datasets DE Q30-3 images and VMSI between 40–180 keV in steps of 20 keV were generated. From SE datasets I30-3 images were reconstructed. All images were reconstructed with and without iMAR. Objective image quality was analyzed applying density measurements at standardized positions (e.g. trocar tip and liver parenchyma adjacent to the trocar tip) and semi-automated threshold based segmentation. Subjective image quality was performed using semi-quantitative scores. Analyses were performed by two observers.

**Results:**

At the trocar tip quantitative image analysis revealed significant difference in CT numbers between reconstructions with iMAR compared to reconstructions without iMAR for VMSI at lower keV levels (80 and 100 keV; p = 0.03) and DE (p = 0.03). For liver parenchyma CT numbers were significantly higher in VMSI at high keV compared to low keV (p≤0.01). VMSI at high keV also showed higher CT numbers compared to DE and SE images, though not the level of statistical significance. The best signal-to-noise ratio for VMSI was at 80 keV and comparable to DE and SE. Noise was lowest at 80 keV and lower than in DE and SE. Subjective image quality was best with VMSI at 80 keV regardless of the application of iMAR. iMAR significantly improved image quality at levels of 140 keV and 160 keV. Interreader-agreement was good for quantitative and qualitative analysis.

**Conclusion:**

iMAR improved image quality in all settings. VMSI with iMAR provided metal artifact reduction and better image quality at 80 keV and thus could improve the accurate positioning in CT-guided needle biopsy. In comparison, DE imaging did not improve image quality compared to SE.

## Introduction

The histological collection of samples is crucial for therapy planning e.g. in HCC. Liver biopsy can be guided by ultrasound, computed tomography (CT) or magnetic resonance imaging (MRI). MRI has a limited availability and CT is preferred over ultrasound especially for difficult accesses for local therapy (obese patients, long distance, small lesion diameter, subdiaphragmatic localization or subcostal access). Non-contrast CT is standard for liver biopsy as contrast agent does not obtain better results though the visualization improves pro tempore, but diminishes in the late phase [1]. Accuracy of CT-guided biopsy of the liver, yielding a histologic diagnosis, was between 86%– 98% (sensitivity 81.6–93.5% and specificity 95.5-100%) [1–3].

Metal artifacts represent a significant limitation of the visibility of liver lesions during CT-guided biopsy. It has been reported that the visualization is fourfold insufficient, particularly for small liver lesions < 3 cm diameter [1]. Consequently, false-negative biopsy results rise by 12.8% compared to lesions with good visualization [1]. Metal artifacts are comprised on the one hand of photon starvation artifacts caused by strong absorption and insufficient photon transmission and on the other hand of beam hardening artifacts due to additional absorption of low energy photons [4]. The severity of artifacts is dependent on the metal alloy itself, its thickness, orientation and CT acquisition parameters like tube voltage, tube current and collimation [5]. Imaging the biopsy trocar without the cannula has been reported to mitigate metal artifacts in a phantom model and the cannula can be safely removed during imaging procedure [6].

VMSI is based on dual-energy acquisitions with two different mean photon energies. The resulting X-ray spectra are used to calculate the density for each voxel and to extrapolate it to a certain electron-volt energy level [7]. Thus, VMSI exploits different polychromatic X-ray spectra and depicts images as they had been acquired with one monoenergetic X-ray beam [7, 8]. The advantage of VMSI has been proven for clinical settings in musculoskeletal imaging to reduce metal artifacts [9], in pedicle screw implants [10], but also for visibility of hypovascular hepatic metastases [7, 11]. Signal-to-noise ratio (SNR) for VMSI is not necessary improved in comparison to linearly blended images, though within a certain keV range VMSI and VMSI+ was better for several applications [12, 13].

Recently new dedicated metal artifact reduction postprocessing algorithms have been developed to further diminish metal artifacts and thus improving the visualization of the biopsy target. In this study, the applied algorithm is based on normalized metal artifact reduction (NMAR) and frequency split metal artifact reduction (FSMAR) using an iterative sinogram impainting approach [14, 15]. The application of these algorithms has already been shown for several clinical settings such as total hip placement, spinal hardware and dental prostheses [16–18].

Aim of the study was to evaluate the feasibility of VMSI during CT-guided needle biopsy and to figure out the optimal keV level for metal artifact reduction and optimal image quality. With respect to CT-guided biopsies no work has been published on VSMI and iMAR.

## Materials and methods

Approvals of the regional and institutional veterinary committees were obtained. The ethics committee also approved this study.

### Animal preparation

In an animal model six pigs with body weights ranging from 31–40 kg were examined during CT-guided biopsy. To reduce breathing artifacts animals were intubated, for medication access a central venous catheter in the superior vena cava was used. The induction of anesthesia was performed with intravenous azaperone (6 mg/kg; Stresnil^®^, Janssen Animal Health, Beerse, Belgium), midazolam (0.4 mg/kg; Dormicum^®^, Roche, Basel, Switzerland), and ketamine (8–10 mg/kg; Ketanest-S^®^, Pfizer, Berlin, Germany) and muscle relaxation and thus respiratory control with vecuronium (0.02 mg/kg; Vecuronium Inresa^®^, Inresa, Freiburg, Germany) was given. The anesthesia was maintained with sevoflurane (Sevofluran Baxter, Baxter, Unterschleißheim, Germany).

One investigator with five years of experience in abdominal and interventional radiology performed all biopsies. A commercially available biopsy needle was used (Spirotome^®^, Bioncise, Hasselt, Belgium) and positioned into the right liver lobe in end-expiratory breath hold and in a standardized manner. The trocar had a length of 14.5 cm and a shaft diameter of 13-gauge. The needle itself (22 cm length, 14-gauge) had a helically configured tip to obtain a controlled histological sample. During imaging the cannula was removed to mitigate artifacts. A coplanar puncture direction to the axial plane was aimed at.

### CT protocol and image reconstructions

#### CT acquisitions

CT acquisitions were obtained immediately after trocar placement on a 64 row, dual-energy CT scanner (Somatom^**®**^ Definition Flash, Siemens Healthineers, Forchheim, Germany). Each animal was scanned with single-energy (SE) and dual-energy (DE) settings as followed for single- energy 120 kV_p_/200 mAs and for dual-energy Sn140 kV_p_ (135 mAs) and 100 kV_p_ (175 mAs). An additional 0.1 mm tin filter was applied to harden the 140 kV_p_ spectrum. The radiation dose of dual-energy examination (CTDI_vol_ = 13.47 mGy) was equalized to a routine single-energy examination with 120 kV_p_ (CTDI_vol_ = 13.40 mGy). Tube current modulation was switched off for single-energy and dual-energy acquisitions. Collimation was 64 x 0.6 mm, pitch 0.6, gantry rotation time 0.28 s and scan length was kept constant for all animals. Scan direction was craniocaudal.

#### Reconstructions

**Fig 1:**
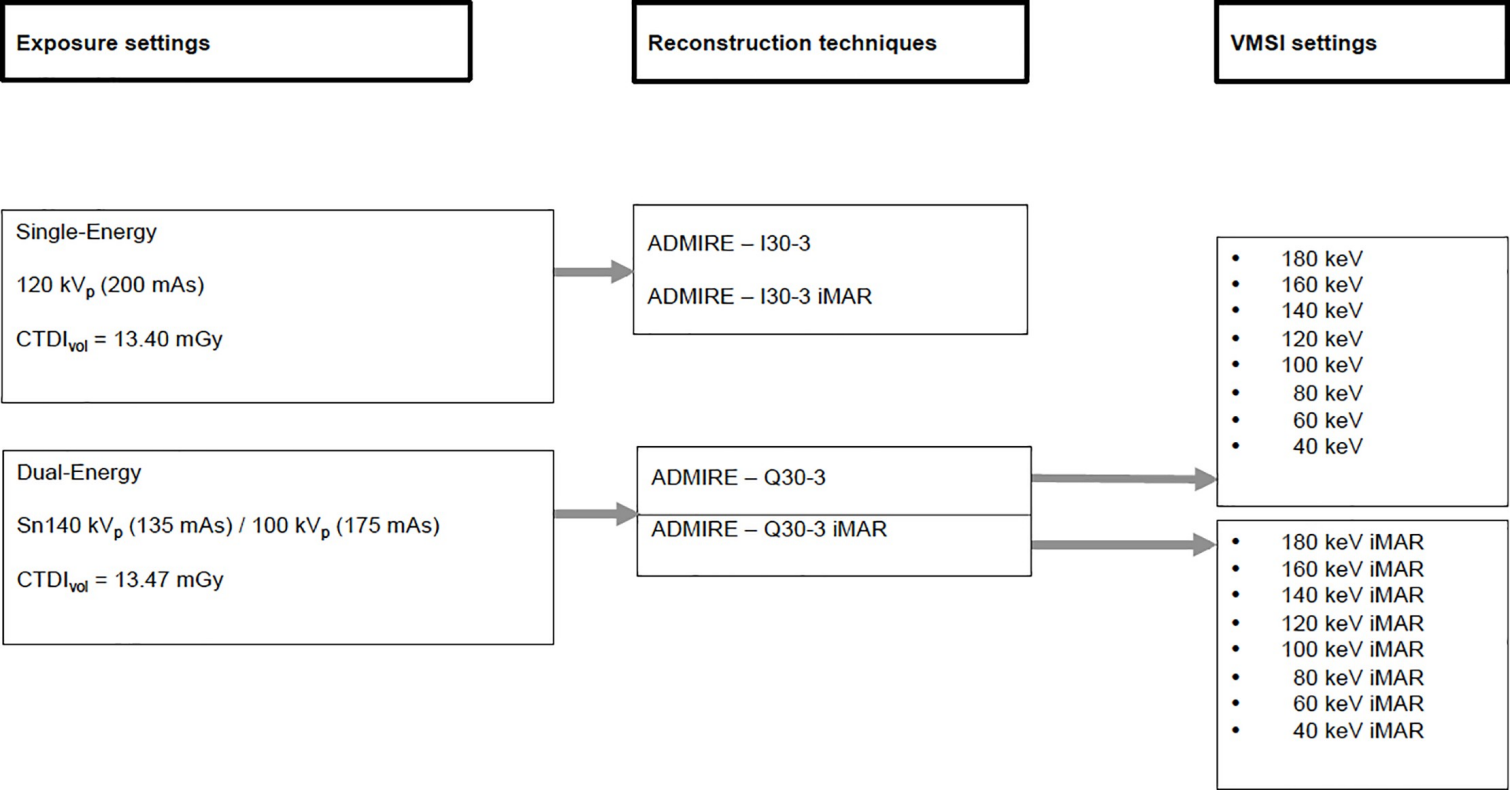
CT acquisition parameters and reconstruction settings.

Reconstructions with single-energy iterative algorithm ADMIRE-I30-3 and dual-energy iterative algorithm ADMIRE-Q30-3 were performed for all energy levels **(Fig 1)**. A soft kernel was selected as the main window setting as during puncture procedure a soft kernel is needed to evaluate further proceedings.

Linear mixed images composed of 140 kV_p_ and 100 kV_p_ data with a mixing ratio of M0.5 were generated. Postprocessing software (Syngo dual energy, Siemens Healthineers, Erlangen, Germany) was used to generate VMSI images. Further, virtual monochromatic spectral images were reconstructed without and with iMAR from the acquired scan with the tube voltage/current constellation (Sn140/100 kV_p_) and reconstruction ADMIRE-Q30-3. VMSI were calculated for energy levels from 40 keV to 180 keV in steps of 20 keV.

Consequently, for the VMSI 16 reconstructions (8 reconstructions with iMAR and 8 reconstructions without iMAR) were performed for image analysis from the linearly blended images with mixed ratio of M0.5, 2 polychromatic linearly blended dual-energy reconstructions with and without iMAR and 2 polychromatic single-energy reconstructions with and without iMAR as well (**Fig 1**). Reconstruction orientation was axial with identical field of view. A standard slice thickness of 2 mm and increment of 1 mm was chosen.

### Image analysis

Objective quantitative analyses were performed by two independent observers with 5 and 6 years of experience in abdominal and interventional radiology. Subjective qualitative analyses were performed twice by two independent observers.

#### Quantitative image analysis

For objective quantitative evaluation data sets were relocated on a multi-modality workstation (syngo.via, Siemens Healthineers, Erlangen, Germany). Four Regions Of Interest (ROI) were analyzed as follows: in the slice with the maximum beam hardening artifacts (a) tip of the trocar, (b) liver parenchyma adjacent to the trocar tip in puncture direction; in a slice without any visible artifacts (c) inferior vena cava serving as a correlate for a hypodense liver lesion and (d) liver parenchyma, which is used to calculate Signal-to-Noise-Ratio (SNR) and image noise. To ensure comparability of measurements ROI area was kept at 0.5 cm^2^ ± 0.04 cm^2^ as bigger ROI was limited by the size of the inferior vena cava. The ROI from one data set were transcribed to other reconstructions within one animal enabling an exact intra-individual comparison. Mean and standard deviation of the density measurements in Hounsfield Units (HU) were extracted. The datasets were evaluated twice.

**Fig 2:**
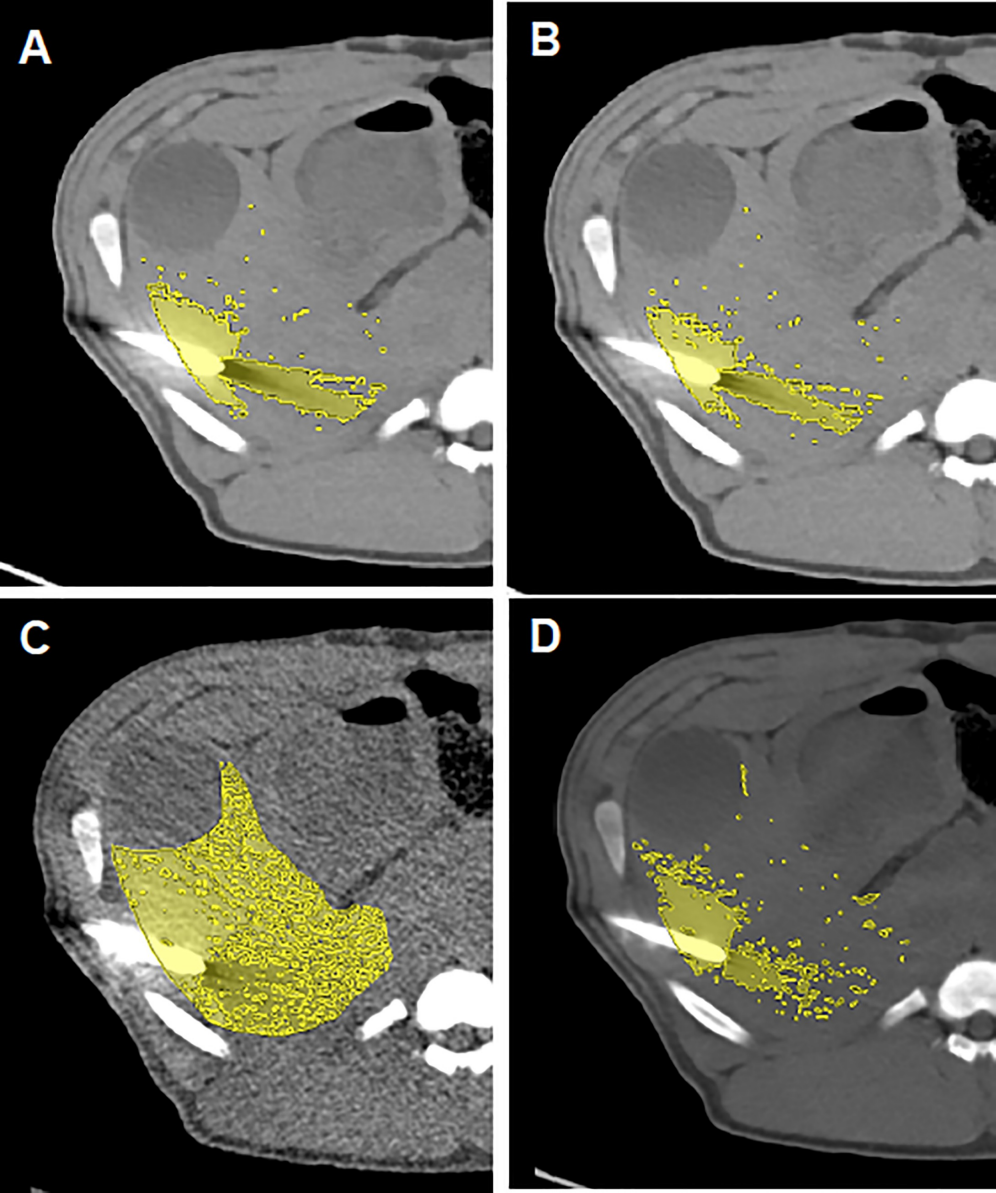
Artifacts based on semi-automated segmentation for SE I30-3 with iMAR (A) and without iMAR (B), VMSI 40 keV iMAR (C) and VMSI 80 keV iMAR (D).

Additionally, artifacts of the series SE, DE, VMSI 40 keV, 80 keV, 180 keV with and without iMAR were quantitatively analyzed for all animals with a semi-automatic segmentation based tool. For the determination of a reference level and calculation of thresholds the liver without any visible artifacts was segmented on native axial slices **(Fig 2)**. The segmentation was performed with the Medical Imaging Interaction Toolkit and with a threshold based semi-automated segmentation method [19, 20]. Artifacts within the liver were segmentated based on the thresholds on the axial slice with the biggest extent of photon starvation artifact bordering the trocar tip. Percentage of artifact volumes in comparison to the segmentated liver area were calculated.

#### Qualitative image analysis

**Fig 3:**
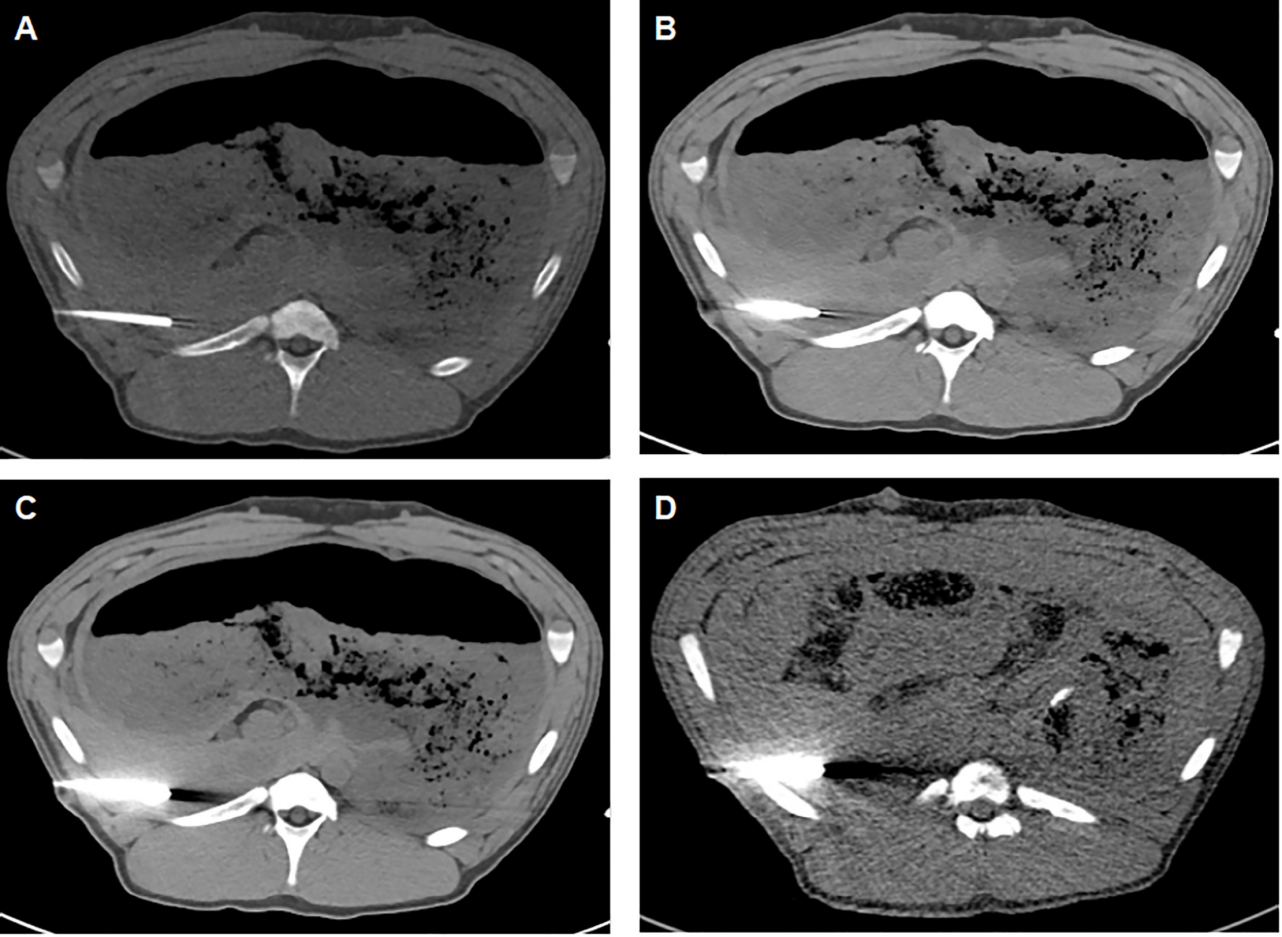
Examples of qualitative image analysis. Semi-quantitative score for artifacts bordering the trocar tip (A: none; B: mild; C: moderate; D: severe).

For subjective qualitative evaluation data sets were viewed on a standard diagnostic working station (Picture Archiving and Communication System [PACS] Centricity, GE, Boston, USA). Width/center of 400/40 HU was chosen as a preset, as the most interesting window during the puncture procedure the biopsy needle and liver parenchyma is the soft tissue kernel window. The datasets were randomized, blinded and evaluated by both observers twice. Observers were allowed to freely scroll through the whole dataset and adapt window settings. Overall image quality was rated on a 5-point Likert-scale (1-excellent; 2-good; 3-fair; 4-poor; 5-non-diagnostic) taking the delineation of the inferior vena cava and aorta as well as image noise into consideration. The extent of artifacts in the liver parenchyma bordering the trocar tip was evaluated on a 5-point Likert-scale (1-none; 2-mild; 3-moderate; 4-severe; 5-non-diagnostic) **(Fig 3)**. Regarding the evaluation of new artifacts generated by iMAR datasets were unblinded concerning with or without iMAR. The artifacts evaluated on a 5-point Likert-scale (1-none; 2-peripheral or around the trocar; 3-peripheral and around the trocar; 4-severe; 5-non-diagnostic).

### Statistical analysis

Statistics was performed using Prism (Prism, 7.0b 2016, GraphPad Software, La Jolla, USA). A non-normal distribution of the data was expected. The level of significance was set at p<0.05. The p-value was used descriptively and a 95% confidence interval was accepted. For the Cohen’s kappa analysis XLSTAT (XLSTAT Version 2018.6, ADDINSOFT 2018, Paris, France) was used.

#### Quantitative image analysis

The Wilcoxon matched-pairs signed rank test was applied to compare non-iMAR images and iMAR images for each monochromatic energy level. Different monochromatic energy levels and linearly blended M0.5 polychromatic DE (140 kV_p_/200 mAs) and SE (120 kV_p_/200 mAs) were analyzed using Friedman test.

#### Qualitative image analysis

Evaluations of qualitative analysis were tabulated according energy levels and reconstruction algorithms. Monochromatic and polychromatic energy levels were analyzed with Friedman test and reconstruction algorithms (iMAR vs. non-iMAR) with paired Wilcoxon t-test.

#### Interreader-agreement

The interreader-agreement of the objective quantitative image analysis was performed with Bland-Altman analysis [21, 22]. The interreader-agreement of the subjective qualitative image analysis was rated with Cohen’s kappa coefficient and graded using the Landis and Koch classification [23, 24].

## Results

### Quantitative image analysis

#### Density measurements

**Fig 4:**
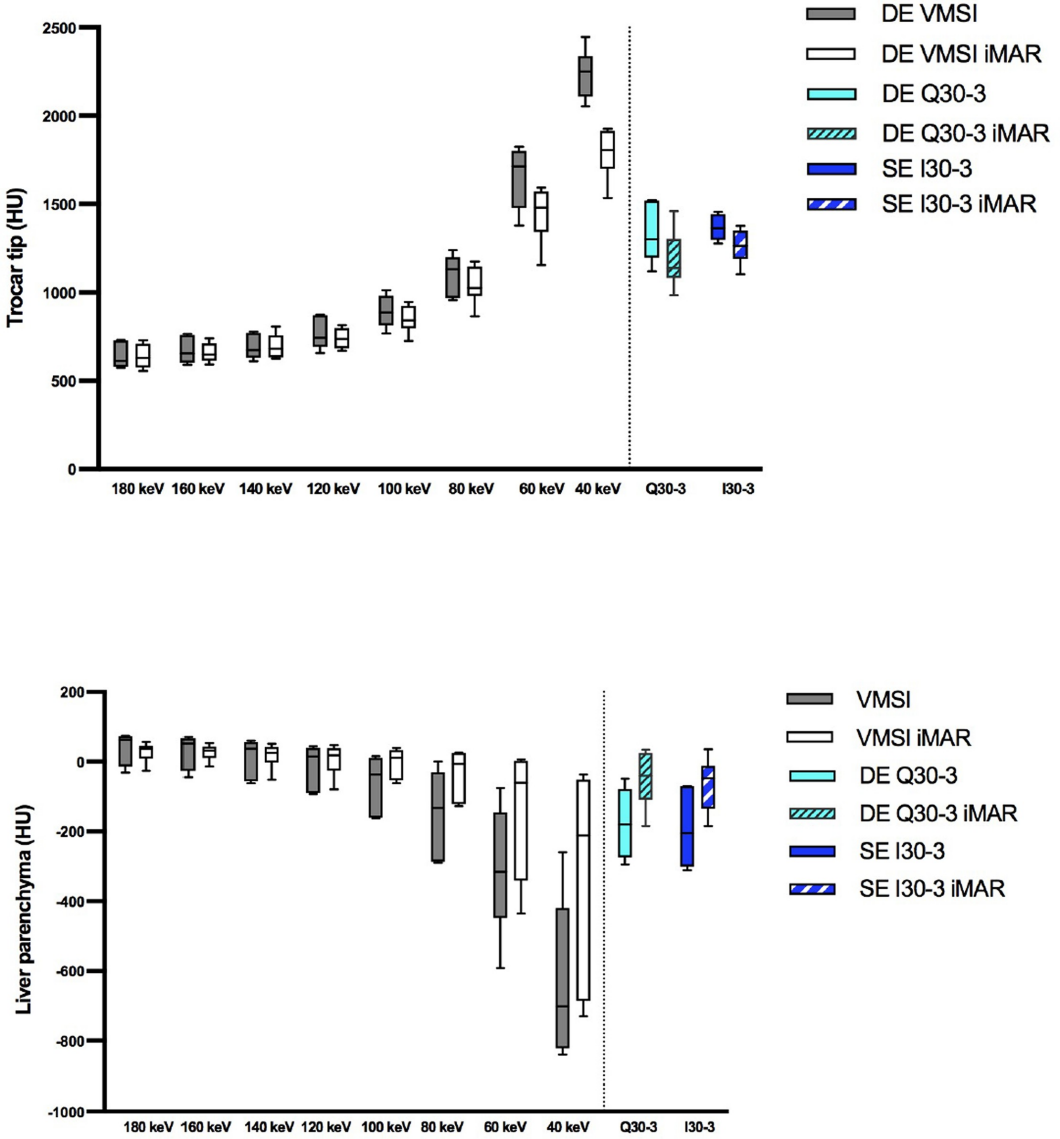
**A.** HU values at the trocar tip for VMSI at different keV levels, linearly blended dual-energy images (Q30-3) and single-energy images (I30-3) with and without iMAR. **B.** HU values in the liver parenchyma adjacent to the trocar tip for VMSI at different keV levels, linearly blended dual-energy images (Q30-3) and single-energy images (I30-3) with and without iMAR.

At the trocar tip with VMSI iMAR only showed significant effects for lower energy levels from 40 keV to approximately 80 keV or 100 keV **(Tables 1 and 2 and S1 Table; Fig 4A)**. In linearly blended DE Q30-3 iMAR showed significant differences in contrast to SE I30-3. High keV levels showed lower HU values in comparison to lower keV levels and DE Q30-3 and SE I30-3 irrespective of iMAR. At liver parenchyma adjacent to the trocar tip iMAR significantly increased HU values of lower VMSI levels, DE Q30-3 and SE I30-3 (p = 0.03) **(Fig 4B)**. Within a reconstruction algorithm irrespective of iMAR high keV levels showed higher HU values in comparison to lower keV levels and DE Q30-3 and SE I30-3.

**Table 1:** Crosstable for p-values based on HU measurements at the trocar tip and liver parenchyma adjacent to the trocar tip, SNR and noise on axial images with VMSI, SE I30-3 and DE Q30-3 without iMAR.

	SE non-iMAR				DE (M0.5) non-iMAR			
	Trocar tip	Liver parenchyma adjacent to the trocar tip	SNR	Noise	Trocar tip	Liver parenchyma adjacent to the trocar tip	SNR	Noise
**180 keV**	p<0.01	p<0.01	p<0.05	p<0.05	p<0.01	p<0.01	x	x
**160 keV**	x	x	p<0.01	p<0.05	x	x	x	x
**140 keV**	x	x	x	x	x	x	x	x
**120 keV**	x	x	x	x	x	x	x	x
**100 keV**	x	x	x	x	x	x	x	x
**80 keV**	x	x	x	x	x	x	x	x
**60 keV**	x	x	x	x	x	x	x	x
**40 keV**	x	x	p<0.0001	p<0.0001	x	x	p<0.01	p<0.01
**DE (M 0.5)**	x	x	x	x	--	--	--	--

**Table 2:** Crosstable for p-values based on HU measurements at the trocar tip and liver parenchyma adjacent to the trocar tip, SNR and noise on axial images with VMSI, SE I30-3 and Q 30–3 with iMAR.

	SE iMAR				DE (M0.5) iMAR			
	Trocar tip	Liver parenchyma adjacent to the trocar tip	SNR	Noise	Trocar tip	Liver parenchyma adjacent to the trocar tip	SNR	Noise
**180 keV iMAR**	p<0.01	p<0.05	p<0.05	p<0.05	p<0.05	x	x	x
**160 keV iMAR**	x	x	x	x	x	x	x	x
**140 keV iMAR**	x	x	x	x	x	x	x	x
**120 keV iMAR**	x	x	x	x	x	x	x	x
**3keV iMAR**	x	x	x	x	x	x	x	x
**80 keV iMAR**	x	x	x	x	x	x	x	x
**60 keV iMAR**	x	x		x	x	x	x	x
**40 keV iMAR**	x	x	p<0.001	p<0.001	x	x	p<0.01	p<0.01
**DE (M 0.5)**	x	x	x	x	--	--	--	--

In the artifact-free slice there were no significant differences in HU values in non-iMAR images vs. iMAR images for liver parenchyma (p = 0.09–0.99) and inferior vena cava (p = 0.13–0.99) in any acquisition and reconstruction algorithm **(S3 Table)**. No significant differences were found between the different VMSI energy levels in the artifact-free slice. No significant differences were found between SE I30-3 and DE Q30-3.

#### Volume percentage of artifacts

Applying the semi-automated segmentation based evaluation of artifacts percentage within the liver iMAR images (0.21–0.78%) showed significantly less artifacts than non-iMAR images (0.26–0.81%) for SE I30-3, DE Q30-3, VMS 40 keV and VMS 80 keV (p = 0.03) **(Table 3**). iMAR showed no significant metal artifacts reduction on the 180 keV images (p = 0.16). The lower the keV level the more artifacts were seen irrespective of iMAR. There was no statistically significant difference in artifact volume between DE acquisition (0.43%) in comparison to SE (0.26%) was observed. 80 keV (non-iMAR: 0.33% and iMAR:0.24%) showed no significant difference in artifact extent in comparison to SE (0.26% and 0.21%, respectively) and DE (0.43% and 0.27%, respectively).

**Table 3:** Results of threshold based semi-automated segmentation, given as a percentage of the liver.

	non-iMAR	iMAR	p-value
**180 keV**	0.25 (0.21; 0.28)	0.26 (0.23; 0.32)	0.1562
**80 keV**	0.33 (0.27; 0.49)	0.24 (0.17; 0.38)	**0.0312**
**40 keV**	0.81 (0.77; 0.88)	0.78 (0.71; 0.82)	**0.0312**
**DE Q30-3 (M 0.5)** Sn140/100 kV_p_	0.43 (0.3; 14)	0.27 (0.22; 0.44)	**0.0312**
**SE I30-3** 120 kV_p_	0.26 (0.23; 0.44)	0.21 (0.19; 0.39)	**0.0312**
**p-value**	**0.0006**^**1**^	**0.0046**^**2**^	

Dunn’s test for multiple comparisons

^1^: I30-3 13.5 mGy vs. 40 keV: p = 0.0191

Q30-3 13.5 mGy vs. 180 keV: p = 0.0349

40 keV vs. 180 keV: p = 0.0013

^2^: I30-3 13.5 mGy iMAR vs. 40 keV iMAR: p = 0.0191

40 keV iMAR vs. 80 keV iMar: p = 0.0052

#### Image noise

**Fig 5:**
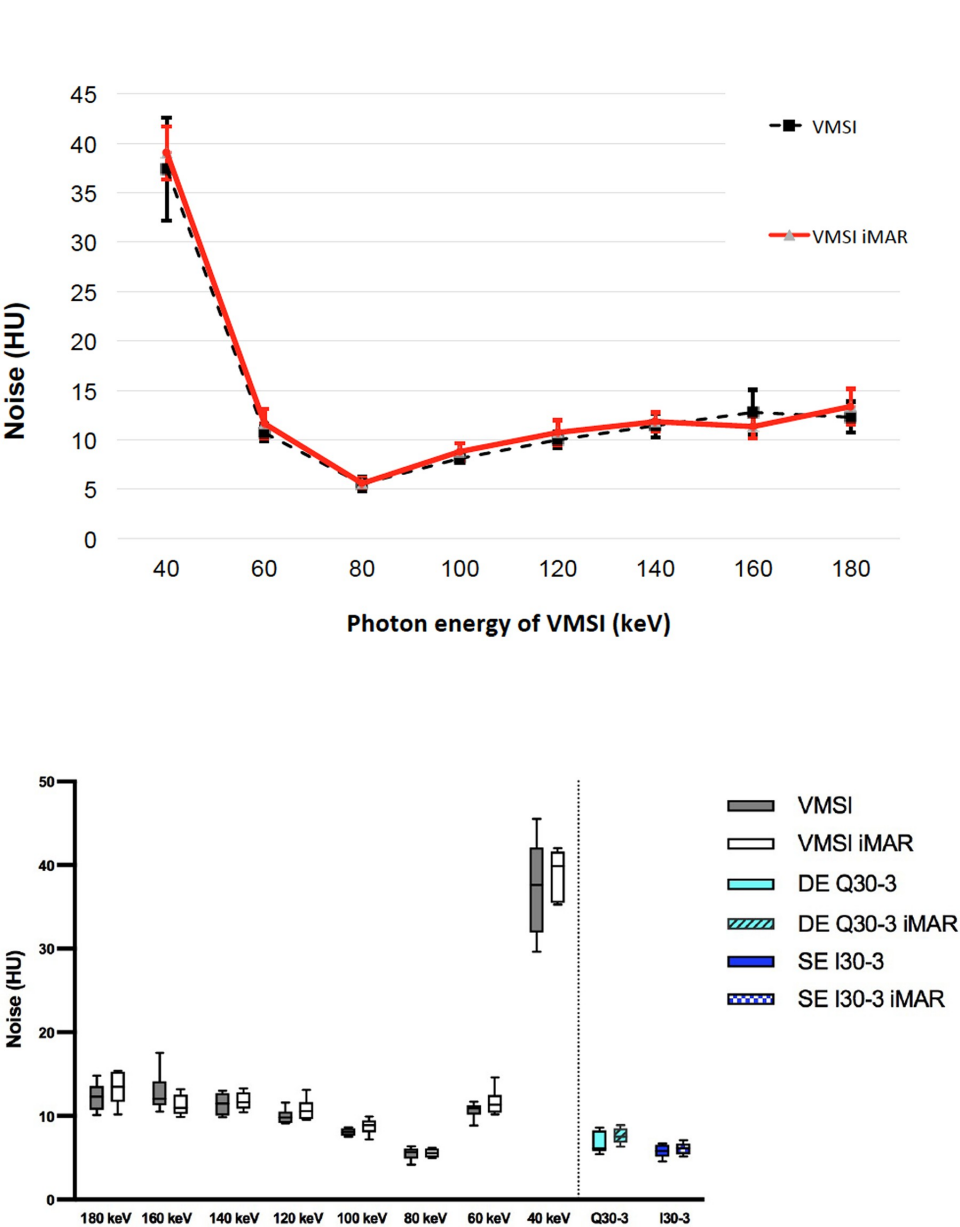
**A.** Mean noise of the liver parenchyma on VMSI for each monochromatic level (keV) without iMAR (black dotted line) and without iMAR (red line) with standard deviation as error bars considering the axial slice without visible artifacts. **B.** Noise of the liver parenchyma on VMSI for each monochromatic level (keV) in the axial slice without visible artifacts.

No significant differences were found between iMAR and non-iMAR images **(Tables 1 and 2 and S4 Table).** Noise was lowest at 80 keV irrespective of iMAR (**Fig 5A and 5B**). Significant differences were found for 80 keV vs. other keV levels (non-iMAR: p = 0.0001–0.0425; iMAR: p = 0.001–0.0269).

#### SNR

**Fig 6:**
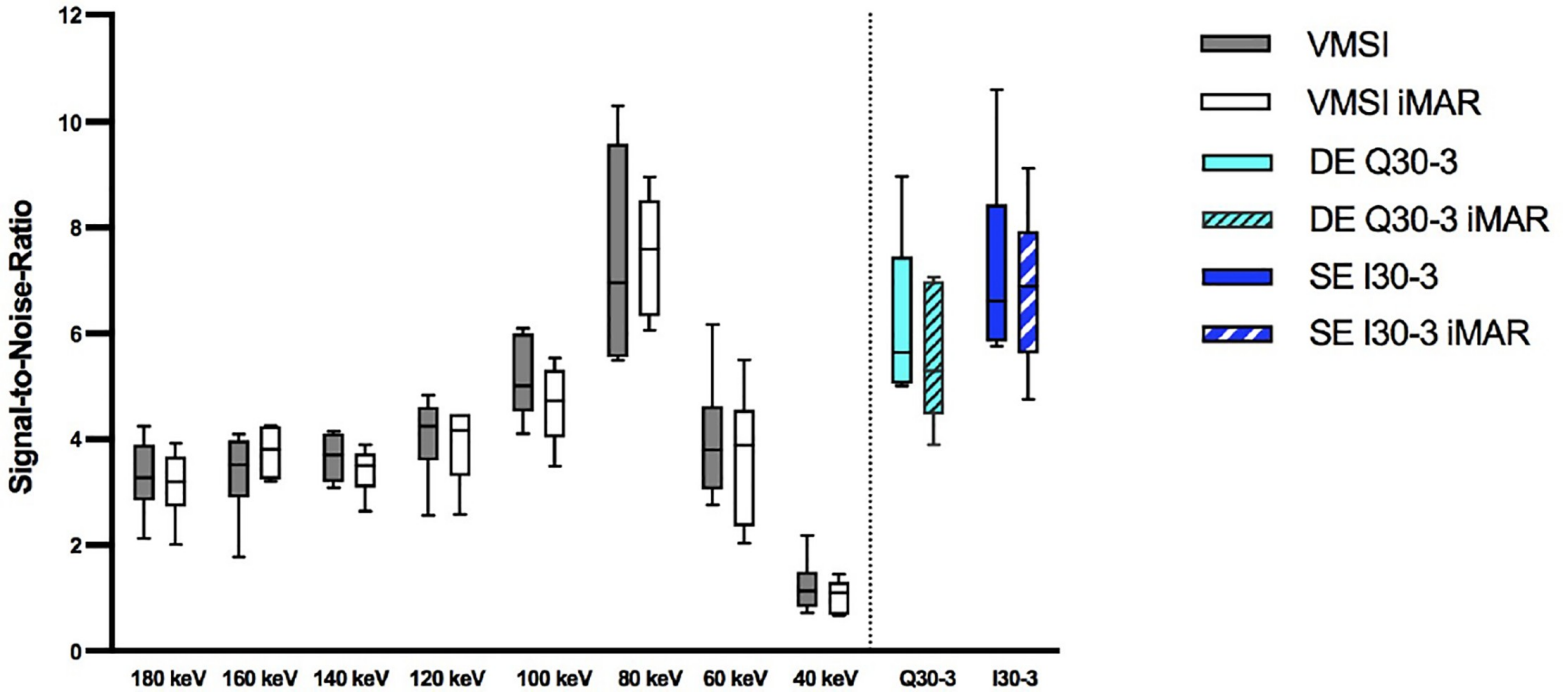
SNR considering the axial slice without artifacts.

SE I30-3 showed high SNR (6.61–6.89). Decreased SNR was found for linearly blended DE reconstructions Q30-3 (5.29–5.64) **(S5 Table and Fig 6).** Best SNR altogether and within VMSI was seen at 80 keV irrespective of iMAR (6.96–7.60). No difference in SNR was found between iMAR and non-iMAR images.

### Qualitative image analysis

#### Overall image quality

**Fig 7:**
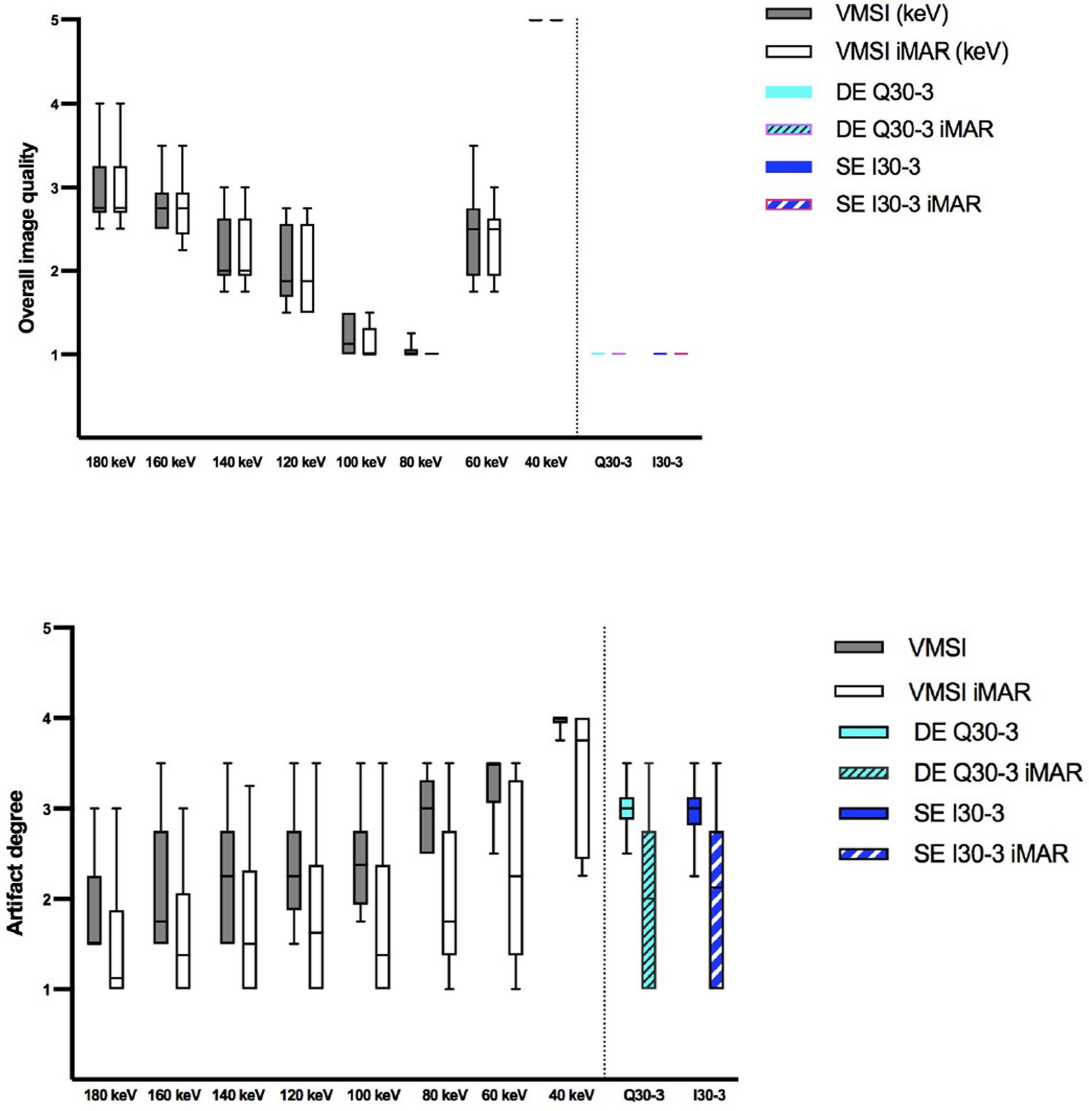
Results qualitative image analysis: Overall image quality (A) and artifact degree in the liver parenchyma bordering the trocar tip (B). Overall image quality was best for VMSI at the level of 80 keV comparable to linearly blended DE Q30-3 and SE I30-3. The artifact degree is significant lower for iMAR images and increases with the VMSI energy level, but comparable to DE Q 30–3 and SE I30-3 at 80–100 keV.

There are no differences in overall image quality between iMAR and non-iMAR images **(Fig 7A)**. Overall image quality was best in VMSI at 80 keV and 100 keV. In comparison to VMSI, SE I30-3 and linearly blended DE Q30-3 images had a better overall image quality in general. However, the best VMSI reconstructions at 80 keV were not significant different from SE or DE images.

#### Artifacts in liver parenchyma in extension of the trocar tip

Artifacts around the trocar tip were less with iMAR for all acquisition modes and reconstruction algorithms (**Fig 7B**), whereby significance was found at 140 and 160 keV. For VMSI the lower the keV the more artifacts were found and thus the artifact degree was negatively correlated to the keV level. The artifact degree at 80 keV were comparable to SE and DE data.

#### New artifacts generated by iMAR

On most images new artifacts could be found around the trocar or/and at the image periphery but none at the trocar tip in puncture direction for VMSI, SE and DE data. The new artifacts around the trocar create an irregular contour due to blooming artifacts. Artifacts in the image periphery are presented as slight dark streaks.

#### Interreader-agreement

The interreader-agreement for the quantitative analysis with the Bland-Altman-analysis is shown in **S6 Table** and shows a good agreement taking into consideration the big variation range of the density measurements, especially at the maximum starvation artifact and liver parenchyma bordering the trocar.

The interreader- for the qualitative analysis was substantial with a Cohen’s Kappa coefficient at 0.877 according to the classification of Landis and Koch.

## Discussion

The overarching goal of this study was to evaluate the use of VMSI and iMAR during CT-guided biopsy. The CTDI_vol_ was matched during acquisition at different tube voltages and tube currents in single-energy CT and dual-energy CT. This enabled a direct side-by-side comparison of image noise and image quality quantitatively and qualitatively. The advantage of VMSI was that high-energy images could be generated without using additional radiation dose as tube currents could be equaled to a routine single-energy examination. Before, in clinical practice an increase of tube voltage settings was performed to mitigate metal artifacts to produce high energy photons for improved metal implant transversion. This resulted in an immense nonlinear increase of radiation dose for the patient.

### Metal artifacts

It is important to differentiate metal artifacts during CT-guided biopsies in beam hardening artifacts at the trocar tip and photon starvation artifacts in the liver parenchyma as CT numbers diverge into the opposite direction. High CT numbers is observed at the trocar tip and decreased when applying dedicated metal artifact reduction assuming that blooming and beam hardening artifacts were diminished. In contrast, CT numbers increased in the liver parenchyma bordering to the trocar tip when iMAR was performed due to less photon starvation artifacts. With regard to iMAR metal artifacts were significantly reduced independent of the mode of acquisition SE, linearly blended DE or mode of reconstructions at different VMSI energy levels. Moreover, iMAR was more effective in lower VMSI energy levels. Though noise was higher in VMSI similar to Mangold et al. the amount of metal artifacts was significantly lower for VMSI extrapolated images at higher keV levels [29]. The assumption of this effect was that more photons pass through the implant and contribute to image generation and hence reduced noise. Bamberg et al. suggested a range of 95–150 keV for prosthesis dependent on prosthesis alloy and body diameter [9]. The metallic biopsy needle itself was best depicted at high energy levels, but for puncture procedures the optimal keV setting of 80 keV was to reduce photon starvation artifacts, which impeded the accurate needle positioning. In comparison to SE acquisition DE did show reduced metal artifacts at the trocar tip or the bordering liver parenchyma, though not statistically significant.

The results of the semi-automated segmentation evaluation are in line with the ROI evaluation. iMAR showed significant metal artifact reduction. No advantage of DE acquisition in comparison to SE was observed. 80 keV showed statistically comparable artifact extent in comparison to SE and DE irrespective of iMAR. For the evaluation based on semi-automatic segmentation with the help of thresholds only one observer is needed. The disadvantage of this method is the inclusion of the complete trocar into artifact volume calculation. However, this does not influence the comparison between reconstruction algorithms. The ROI-analysis might be more precise in the differentiated evaluation of the metal artifacts as artifacts due to beam hardening and photon starvation.

### Image quality

The quantitative results showed that image quality criteria (i.e. noise and SNR) were slightly better in single-energy compared to linearly blended dual-energy, though not significant in the qualitative evaluation, which was in line with the previous study from Nattenmueller et al. [25]. Higher noise was seen in low and also in high keV range and reported by Zhang et al. and Xia et al. [26, 27]. This study confirmed results from other previous phantom and clinical studies showing that lowest image noise of the liver parenchyma had been yielded at approximately 70 keV [11, 28]. Though in this study noise was higher in most of VMSI images compared to solely single-energy and dual-energy data still noise was lowest at 80 keV for VMSI, which showed comparable values quantitatively and qualitatively to linearly blended DE and SE. We also noted that noise was not affected by using iMAR. Similar observations concerning noise were also found for SNR. SNR was lower for most VMSI than SE and DE, except VMSI at energy level of 80 keV SNR was even better than the polychromatic images of SE and linearly blended DE.

### Limitations

Importantly, some limitations of this study have to be mentioned. Dual-energy acquisition and VMSI is not available everywhere and we did not compare iterative metal artifact reduction algorithms from different vendors. Another point was the impact of the trocar angle on the extent of metal artifacts. Though we did not expect this to affect the comparison of different algorithms as the trocar angle remained unchanged intra-individually, this could be further evaluated in specific clinical trials.

A trade-off had to be made between reducing metal artifacts by high tube voltage CT acquisition and thus, improving image quality for puncture guidance on the one hand and reducing radiation dose on the other hand. This study indicated same as Pessis et al. that the advantage of DE was the possibility to generate VMSI without additional radiation dose [30]. Moreover, the use of DE alone did not improve metal artifact reduction, but virtual monochromatic extrapolation based on dual-energy CT yielded metal artifact reduction and provided the best image quality. In the special case of CT-guided biopsies a keV level of 80 keV could be recommended. The limitation of this study in an animal model instead of human subjects was owed to the fact that CT acquisition of the same body region with different exposure settings would have resulted in a high overall radiation dose. Also the number of subjects was limited and produced a lager standard error and wider confidence interval than higher animal numbers.

## Conclusion

Dual-energy alone did not reduce metal artifacts sufficiently, but VMSI based on dual-energy CT could significantly mitigate metal artifacts during CT-guided biopsy providing improved depiction of liver lesions and a safe and versatile access for long puncture pathways. If available VMSI at 80 keV is recommended. iMAR should deployed to mitigate metal artifacts independently of acquisition mode and reconstruction algorithm.

## Supporting information

S1 Table HU values measured on an axial slice at the trocar tip. (DOCX)

S2 Table HU values in the liver parenchyma adjacent to the trocar tip in puncture direction. (DOCX)

S3 Table HU values for inferior vena cava (IVC) and liver parenchyma considering the axial slice without any visible artifacts. (DOCX)

S4 Table Noise measured in the liver parenchyma on an axial slice without any visible artifacts. Mean noise was lowest at 80 keV. No significant difference between iMAR and non-iMAR images was found.
(DOCX)

S5 Table Signal-to-noise ratio (SNR) was highest for SE I30-3 and VMSI at 80 keV. No significant difference in SNR was found between iMAR and non-iMAR images.
(DOCX)

S6 Table Interreader-agreement with Blant-Altman analysis showing difference and in brackets 95% limits of agreement. (DOCX)
