# Supplementary material for: Virtual monochromatic spectral imaging versus linearly blended dual-energy and single-energy imaging during CT-guided biopsy needle positioning: Optimization of keV settings and impact on image quality
Source: PLoS One. 2020 Feb 10;15(2):e0228578. doi: 10.1371/journal.pone.0228578 (PMC7010258; doi:10.1371/journal.pone.0228578)
Supplement: S1 Table — (DOCX) [file pone.0228578.s001.docx]

**Table 1:** HU values measured on an axial slice at the trocar tip

|  | **non-iMAR** | **iMAR** | **p-value** |
| --- | --- | --- | --- |
| **180 keV** | 613  (580;705) | 630  (586;692) | 0.8438 |
| **160 keV** | 655  (613;739) | 649.35  (622;697) | 0.4375 |
| **140 keV** | 674  (637;755) | 683.33  (639.99;733.34) | 0.8438 |
| **120 keV** | 745  (711;844) | 737  (698;781) | 0.2188 |
| **100 keV** | 887  (836;958) | 843  (819;904) | **0.0312** |
| **80 keV** | 1132  (1008;1179) | 1025  (1019;1110) | 0.0938 |
| **60 keV** | 1713  (1557;1776) | 1481  (1419;1548) | **0.0312** |
| **40 keV** | 2251  (2152;2296) | 1805  (1761;1889) | **0.0312** |
| **DE Q30-3 (M 0.5)**  Sn140/100 kV_p_ | 1301  (1241;1465) | 1139  (1116;1227) | **0.0312** |
| **SE I30-3**  120 kV_p_ | 1363  (1306;1431) | 1264  (1228;1324) | 0.0625 |
| **p-value** | **<0.0001^1^** | **<0.0001^2^** |  |

Dunn’s test for multiple comparisons:

**^1^** I30-3 13.5 mGy vs. 180 keV p-value: 0.0062

Q30-3 13.5 mGy vs. 180 keV p-value: 0.0090

180 keV vs. 60 keV p-value: 0.0003

180 keV vs. 40 keV p-value: <0.0001

160 keV vs. 60 keV p-value: 0.0042

160 keV vs. 40 keV p-value: 0.0002

140 keV vs. 60 keV p-value: 0.0381

140 keV vs. 40 keV p-value: 0.0028

120 keV vs. 40 keV p-value: 0.0269

**^2^** I30-3 13.5 mGy iMAR vs. 180 keV iMAR p-value: 0.0062

Q30-3 13.5 mGy iMAR vs. 180 keV iMAR p-value: 0.0131

180 keV iMAR vs. 60 keV iMAR p-value: 0.0002

180 keV iMAR vs. 40 keV iMAR p-value: <0.0001

160 keV iMAR vs. 60 keV iMAR p-value: 0.0028

160 keV iMAR vs. 40 keV iMAR p-value: 0.0002

140 keV iMAR vs. 60 keV iMAR p-value: 0.0269

140 keV iMAR vs. 40 keV iMAR p-value: 0.0028

120 keV iMAR vs. 40 keV iMAR p-value: 0.0269
