# Supplementary material for: Virtual monochromatic spectral imaging versus linearly blended dual-energy and single-energy imaging during CT-guided biopsy needle positioning: Optimization of keV settings and impact on image quality
Source: PLoS One. 2020 Feb 10;15(2):e0228578. doi: 10.1371/journal.pone.0228578 (PMC7010258; doi:10.1371/journal.pone.0228578)
Supplement: S2 Table — (DOCX) [file pone.0228578.s002.docx]

**Table 2:** HU values in the liver parenchyma adjacent to the trocar tip in puncture direction

|  | **no iMAR** | **iMAR** | **p-value** |
| --- | --- | --- | --- |
| **180 keV** | 62  (9;71) | 37  (25;41) | 0.5625 |
| **160 keV** | 52  (-4;64) | 32.00  (21;39) | >0.9999 |
| **140 keV** | 38  (-34;53) | 26  (15;38) | 0.8438 |
| **120 keV** | 14  (-68;36) | 18.9  (-4;35) | 0.2188 |
| **100 keV** | -37  (-133;2) | 11  (-39;31) | **0.0312** |
| **80 keV** | -133  (-253;-58) | -7  (-98;23) | **0.0312** |
| **60 keV** | -316  (-396;-188) | -61  (-259;-2) | 0.0625 |
| **40 keV** | -702  (-807;-511) | -212  (-573;-79) | **0.0312** |
| **DE Q30-3 (M 0.5)**  Sn140/100 kV_p_ | -180  (-256;-101) | -41  (-80;13) | **0.0312** |
| **SE I30-3**  120 kV_p_ | -204  (-296;-82) | -48  (-106; -27) | **0.0312** |
| **p-value** | **<0.0001^1^** | **<0.0001^2^** |  |

^Dunn’s test for multiple comparisons:^

**^1^** I30-3 13.5 mGy vs. 180 keV p-value: 0.0090

Q30-3 13.5 mGy vs. 180 keV p-value: 0.0090

180 keV vs. 60 keV p-value: 0.0003

180 keV vs. 40 keV p-value:<0.0001

160 keV vs. 60 keV p-value: 0.0042

160 keV vs. 40 keV p-value: 0.0002

140 keV vs. 60 keV p-value: 0.0381

140 keV vs. 40 keV p-value: 0.0028

120 keV vs. 40 keV p-value: 0.0269

**^2^** I30-3 13.5 mGy vs. 180 keV iMAR p-value: 0.0189

180 keV iMAR vs. 60 keV iMAR p-value: 0.0012

180 keV iMAR vs. 40 keV iMAR p-value: <0.0001

160 keV iMAR vs. 60 keV iMAR p-value: 0.0062

160 keV iMAR vs. 40 keV iMAR p-value: 0.0001

140 keV iMAR vs. 40 keV iMAR p-value: 0.0028
