# Supplementary material for: Virtual monochromatic spectral imaging versus linearly blended dual-energy and single-energy imaging during CT-guided biopsy needle positioning: Optimization of keV settings and impact on image quality
Source: PLoS One. 2020 Feb 10;15(2):e0228578. doi: 10.1371/journal.pone.0228578 (PMC7010258; doi:10.1371/journal.pone.0228578)
Supplement: S3 Table — (DOCX) [file pone.0228578.s003.docx]

**Table 3:** HU values for inferior vena cava (IVC) and liver parenchyma considering the axial slice without any visible artifacts

|  | **IVC** | | | **Liver parenchyma** | | |
| --- | --- | --- | --- | --- | --- | --- |
|  | **non-iMAR** | **iMAR** | **p-value** | **non-iMAR** | **iMAR** | **p-value** |
| **180 keV** | 41  (39;43) | 41  (39;44) | 0.1562 | 62  (62;64) | 62  (61;65) | 0.5625 |
| **160 keV** | 43  (38;45) | 43  (41;45) | 0.312 | 63  (62;63) | 65  (61;67) | 0.4375 |
| **140 keV** | 43  (39;45) | 43  (40;43) | 0.4375 | 62  (62;65) | 62  (60;63) | 0.0938 |
| **120 keV** | 40  (39;43) | 41  (39;43) | 0.4375 | 63  (61.69;64.44) | 61  (61;63) | 0.6875 |
| **100 keV** | 40  (37;45) | 42  (39;44) | 0.5625 | 62  (61;63) | 61  (60;66) | 0.8438 |
| **80 keV** | 41  (36;43) | 43  (38;45) | 0.1562 | 61  (60;63) | 61  (60;64) | 0.4375 |
| **60 keV** | 42  (36;44) | 42  (33;44) | >0.9999 | 61  (61;63) | 61  (61;62) | 0.7188 |
| **40 keV** | 41  (37;43) | 41  (31;43) | 0.6875 | 57  (56;63) | 62  (61;63) | >0.9999 |
| **DE Q30-3 (M 0.5)**  Sn140/100 kV_p_ | 41  (37;43) | 42  (38;45) | 0.1250 | 62.13  (60.11;62.79) | 61  (60;62) | 0.6875 |
| **SE I30-3**  120 kV_p_ | 41  (37;43) | 43  (36;44) | 0.5625 | 58  (57;59) | 60  (58;62) | 0.1562 |
| **p-value** | 0.5676 | 0.7616 |  | **0.0086^1^** | 0.2897 |  |

Dunn’s test for multiple comparisons:

**^1^** I30-3 13.5 mGy vs. 160 keV p-value: 0.0452

I30-3 13.5 mGy vs. 140 keV p-value: 0.0189
