# Supplementary material for: Virtual monochromatic spectral imaging versus linearly blended dual-energy and single-energy imaging during CT-guided biopsy needle positioning: Optimization of keV settings and impact on image quality
Source: PLoS One. 2020 Feb 10;15(2):e0228578. doi: 10.1371/journal.pone.0228578 (PMC7010258; doi:10.1371/journal.pone.0228578)
Supplement: S4 Table — Mean noise was lowest at 80 keV. No significant difference between iMAR and non-iMAR images was found. (DOCX) [file pone.0228578.s004.docx]

**Table 4:** Noise measured in the liver parenchyma on an axial slice without any visible artifacts. Mean noise was lowest at 80 keV. No significant difference between iMAR and non-iMAR images was found.

|  | **non-iMAR** | **iMAR** | **p-value** |
| --- | --- | --- | --- |
| **180 keV** | 12.30  (11.11;13.11) | 61.98  (61.21;64.76) | 0.5625 |
| **160 keV** | 12.05  (11.55;12.85) | 10.98  (10.38;12.14) | 0.4375 |
| **140 keV** | 11.48  (10.34;12.50) | 11.65  (11.16;12.48) | 0.1562 |
| **120 keV** | 9.83  (9.35;10.08) | 10.50  (9.68;11.10) | 0.0625 |
| **100 keV** | 8.08  (7.71;8.44) | 8.90  (8.44;9.25) | 0.2188 |
| **80 keV** | 5.68  (5.20;5.96) | 5.58  (5.08;6.04) | 0.9062 |
| **60 keV** | 10.93  (10.64;11.06) | 11.35  (10.58;11.83) | 0.1875 |
| **40 keV** | 37.65  (33.63;40.44) | 39.88  (36.48;41.21) | 0.6875 |
| **DE Q30-3 (M 0.5)**  Sn140/100 kV_p_ | 6.10  (5.96;7.66) | 7.50  (7.00;8.26) | 0.0938 |
| **SE I30-3**  120 kV_p_ | 5.83  (5.40;6.40) | 6.13  (5.60;6.54) | 0.4688 |
| **p-value** | **<0.0001^1^** | **<0.0001^2^** |  |

Dunn’s test for multiple comparisons:

**^1^**  I30-3 13.5 mGy vs. 180 keV p-value: 0.0157

I30-3 13.5 mGy vs. 160 keV p-value: 0.0131

I30-3 13.5 mGy vs. 40 keV p-value: <0.0001

Q30-3 13.5 mGy vs. 40 keV p-value: 0.0019

180 keV vs. 80 keV p-value: 0.0157

160 keV vs. 80 keV p-value: 0.0131

140 keV vs. 80 keV p-value: 0.0425

100 keV vs. 40 keV p-value: 0.0189

80 keV vs. 40 keV p-value: <0.0001

**^2^** I30-3 13.5 mGy iMAR vs. 180 keV iMAR p-value: 0.0131

I30-3 13.5 mGy iMAR vs. 40 keV iMAR p-value: <0.0001

Q30-3 13.5 mGy iMAR vs. 40 keV iMAR p-value: 0.0028

180 keV iMAR vs. 80 keV iMAR p-value: 0.0042

140 keV iMAR vs. 80 keV iMAR p-value: 0.0269

100 keV iMAR vs. 40 keV iMAR p-value: 0.0269

80 keV iMAR vs. 40 keV iMAR p-value: <0.0001
