# Supplementary material for: Virtual monochromatic spectral imaging versus linearly blended dual-energy and single-energy imaging during CT-guided biopsy needle positioning: Optimization of keV settings and impact on image quality
Source: PLoS One. 2020 Feb 10;15(2):e0228578. doi: 10.1371/journal.pone.0228578 (PMC7010258; doi:10.1371/journal.pone.0228578)
Supplement: S5 Table — No significant difference in SNR was found between iMAR and non-iMAR images. (DOCX) [file pone.0228578.s005.docx]

**Table 5:** Signal-to-noise ratio (SNR) was highest for SE I30-3 and VMSI at 80 keV. No significant difference in SNR was found between iMAR and non-iMAR images.

|  | **non-iMAR** | **iMAR** | **p-value** |
| --- | --- | --- | --- |
| **180 keV** | 3.27  (3.12;3.67) | 3.20  (3.00;3.51) | 0.8438 |
| **160 keV** | 3.52  (3.32;3.85) | 3.81  (3.36;4.17) | 0.3125 |
| **140 keV** | 3.71  (3.30;4.04) | 3.51  (3.30;3.64) | 0.1562 |
| **120 keV** | 4.25  (4.01;4.47) | 4.17  (3.64;4.45) | 0.4375 |
| **100 keV** | 5.01  (4.71;5.77) | 4.72  (4.28;5.17) | 0.2188 |
| **80 keV** | 6.96  (5.86;8.81) | 7.60  (6.56;8.34) | >0.9999 |
| **60 keV** | 3.79  (3.29;4.06) | 3.88  (2.80;4.16) | 0.4375 |
| **40 keV** | 1.13  (0.93;1.24) | 1.10  (0.77;1.23) | 0.2188 |
| **DE Q30-3 (M 0.5)**  Sn140/100 kV_p_ | 5.64  (5.08;6.76) | 5.29  (4.79;6.59) | 0.2188 |
| **SE I30-3**  120 kV_p_ | 6.61  (6.05;7.45) | 6.89  (6.10;7.44) | 0.4375 |
| **p-value** | **<0.0001^1^** | **<0.0001^2^** |  |

Dunn’s test for multiple comparisons:

I30-3 13.5 mGy vs. 180 keV p-value: 0.0269

I30-3 13.5 mGy vs. 160 keV p-value: 0.0090

I30-3 13.5 mGy vs. 40 keV p-value: <0.0001

Q30-3 13.5 mGy vs. 40 keV p-value: 0.0019

160 keV vs. 80 keV p-value: 0.0189

100 keV vs. 40 keV p-value: 0.0269

80 keV vs. 40 keV p-value: 0.0001

^2^ I30-3 13.5 mGy iMAR vs. 180 keV iMAR p-value: 0.0131

I30-3 13.5 mGy iMAR vs. 40 keV iMAR p-value: 0.0001

Q30-3 13.5 mGy iMAR vs. 40 keV iMAR p-value: 0.0028

180 keV iMAR vs. 80 keV iMAR p-value: 0.0042

140 keV iMAR vs. 80 keV iMAR p-value: 0.0269

80 keV iMAR vs. 40 keV iMAR p-value: <0.0001
