# Supplementary material for: Virtual monochromatic spectral imaging versus linearly blended dual-energy and single-energy imaging during CT-guided biopsy needle positioning: Optimization of keV settings and impact on image quality
Source: PLoS One. 2020 Feb 10;15(2):e0228578. doi: 10.1371/journal.pone.0228578 (PMC7010258; doi:10.1371/journal.pone.0228578)
Supplement: S6 Table — (DOCX) [file pone.0228578.s006.docx]

**Table 6:** Interreader-agreement with Blant-Altman analysis showing difference and in brackets 95% limits of agreement

| **Interreader agreement (HU)** | **SE I30-3** | **SE I30-3 iMAR** | **DE Q30-3** | **DE Q30-3 iMAR** | **80 keV** | **80 keV iMAR** |
| --- | --- | --- | --- | --- | --- | --- |
| **Liver parenchyma**  **adjacent to the trocar tip** | -8.43  (-29.19-12.32) | 0.12  (-17.28-17.52) | -1.8  (-17.33-13.73) | -3.62  (-14.35-7.12) | 2.17  ( -9.68-14.01) | 0.80  (-7.28-8.88) |
| **Noise liver parenchyma**  **(slice without any visible artifacts)** | -0.08  (-3.30-3.13) | 0.30  (-2.89-3.49) | -0.48   \| (-2.733-1.767) \| \| --- \| | -0.55   \| (-3.56-2.46) \| \| --- \| | 0.47  (-0.86-1.79) | -0.50   \| (-3.47 - 2.47) \| \| --- \| |
| **SNR** | 0.12   \| (-3.23-3.46) \| \| --- \| | -0.18  (-4.14-3.77) | 0.29   \| (-1.32-1.90) \| \| --- \| | 0.36   \| (-1.79-2.52) \| \| --- \| | -0.60  (-2.37-1.16) | 0.63   \| (-3.41 – 4.66) \| \| --- \| |
